# Preparation and Characterization of Antioxidant Nanoparticles Composed of Chitosan and Fucoidan for Antibiotics Delivery

**DOI:** 10.3390/md12084379

**Published:** 2014-07-31

**Authors:** Yi-Cheng Huang, Rou-Ying Li

**Affiliations:** Department of Food Science, College of Life Science, National Taiwan Ocean University, 2 Pei Ning Road, Keelung 20224, Taiwan; E-Mail: d91548006@ntu.edu.tw

**Keywords:** chitosan, fucoidan, nanoparticle, antioxidant, antibiotic, delivery

## Abstract

In this study, we developed novel chitosan/fucoidan nanoparticles (CS/F NPs) using a simple polyelectrolyte self-assembly method and evaluated their potential to be antioxidant carriers. As the CS/F weight ratio was 5/1, the CS/F NPs were spherical and exhibited diameters of approximately 230–250 nm, as demonstrated by TEM. These CS/F NPs maintained compactness and stability for 25 day in phosphate-buffered saline (pH 6.0–7.4). The CS/F NPs exhibited highly potent antioxidant effects by scavenging 1,1-diphenyl-2-picrylhydrazyl (DPPH), reducing the concentration of intracellular reactive oxygen species (ROS) and superoxide anion (O_2_^−^) in stimulated macrophages. The DPPH scavenging effect of CS/F NPs primarily derives from fucoidan. Furthermore, these CS/F NPs activated no host immune cells into inflammation-mediated cytotoxic conditions induced by IL-6 production and NO generation. The MTT cell viability assay revealed an absence of toxicity in A549 cells after exposure to the formulations containing 0.375 mg NPs/mL to 3 mg NPs/mL. Gentamicin (GM), an antibiotic, was used as a model drug for an *in vitro* releasing test. The CS/F NPs controlled the release of GM for up to 72 h, with 99% of release. The antioxidant CS/F NPs prepared in this study could thus be effective in delivering antibiotics to the lungs, particularly for airway inflammatory diseases.

## 1. Introduction

Pulmonary delivery systems using particulate drug carriers are an attractive modality for treating airway disorders such as cystic fibrosis [1], asthma [2], pulmonary infections [3], pulmonary hypertension [4], and lung cancers [5]. Pulmonary delivery involves providing a direct and local delivery to the lungs, thus achieving both a selective pulmonary effect and a reduction of side effects. Alternatively, pulmonary drug delivery can be achieved by targeting the alveolar region where the drug can be absorbed through a thin layer (0.1 to 0.5 μm thick) of epithelial cells and into the systemic metabolism. This causes enhanced permeability, absence of first-pass metabolism, rapid onset of action, and high bioavailability [6,7]. Therefore, pulmonary drug delivery serves as a means of delivering drugs either systemically or locally and has drawn increasing attention [8].

To achieve efficient pulmonary drug delivery, particulate drug formulation that delivers therapeutic agents to the site of action for lung diseases is necessary [7]. Nanoparticles (NPs) have recently been proposed as valuable carriers for efficiently transporting drugs to the lung epithelium without mucociliary clearance and phagocytic mechanisms [9,10]. Recent and rapid advances in nanotechnology and materials science have resulted in the development of numerous polymers that can be formulated into particulate drug carriers for efficient drug delivery [11,12]. Liposomes that can manipulate release, and target by altering bilayer constituents, have been used in various delivery systems [13]. However, cationic liposomes widely used for gene delivery have been observed to induce oxygen radical-mediated pulmonary toxicity [14]. Biodegradable polyester nanoparticles, such as poly(lactic-co-glycolic acid) (PLGA), have been widely used as delivery vehicles for various drugs and biopharmaceuticals [15,16]. However, PLGA hydrolytically degrades into acid components that are known to induce inflammatory responses [17]. The intratracheal treatment of PLGA microparticles provokes inflammatory responses, evidenced by a large recruitment of white blood cells to the lungs [18]. Therefore, developing novel antioxidant nanoparticles as pulmonary delivery carriers is necessary.

We recently developed chitosan/fucoidan (CS/F) NPs as carriers for drug delivery systems [19,20]. Fucoidan (F), which is extracted from brown seaweed, is a sulfated polyfucose polysaccharide [21,22]. Fucoidan demonstrates significant antioxidant and anti-inflammatory activities, and has received increasing interest in the field of biotechnology [23,24]. Rocha de Souza *et al.* reported that fucoidan obtained from *Fucus vesiculosus* has an inhibitory effect on the formation of hydroxyl radicals and superoxide radicals [25]. Mekabu fucoidan can relieve pulmonary inflammation and downregulate Th2-dominated responses, which might be useful for treating allergic inflammation [26]. Chitosan (CS), a cationic polysaccharide derived from chitin by alkaline deacetylation, is widely used as a carrier to improve and control the release of drugs [27,28]. In addition to being biocompatible and biodegradable by pulmonary lysozyme [29], CS is mucoadhesive [30] and can promote macromolecule permeation through well-organized epithelia [31,32,33]. CS/tripolyphosphate NPs have demonstrated an excellent capacity for protein entrapment and peptide absorption improvement by using several mucosal routes, such as the nasal and ocular routes [31,34].

In the current study, we developed CS/F NPs and evaluated their potential to be carriers for antibiotics delivery. The prepared CS/F NPs were characterized physicochemically using Fourier transfer infrared spectroscopy (FT-IR), transmission electron microscopy (TEM), and dynamic light scattering (DLS). *In vitro* antioxidant and anti-inflammatory activities of CS/F NPs in lipopolysaccharide (LPS)-stimulated macrophages were evaluated by measuring DPPH scavenging abilities, the level of reactive oxygen species (ROS), superoxide anions (O_2_^−^) and inflammatory mediators such as NO and IL-6. The effect of CS/F NPs on macrophages was visualized using inverted fluorescence microscopic imaging and a flow cytometry system. Moreover, the release profile of antibiotics from NP was quantified using spectrophotometric method with gentamicin (GM) as a model drug.

## 2. Results and Discussion

### 2.1. Characterization of CS/F NPs

**Figure 1:**
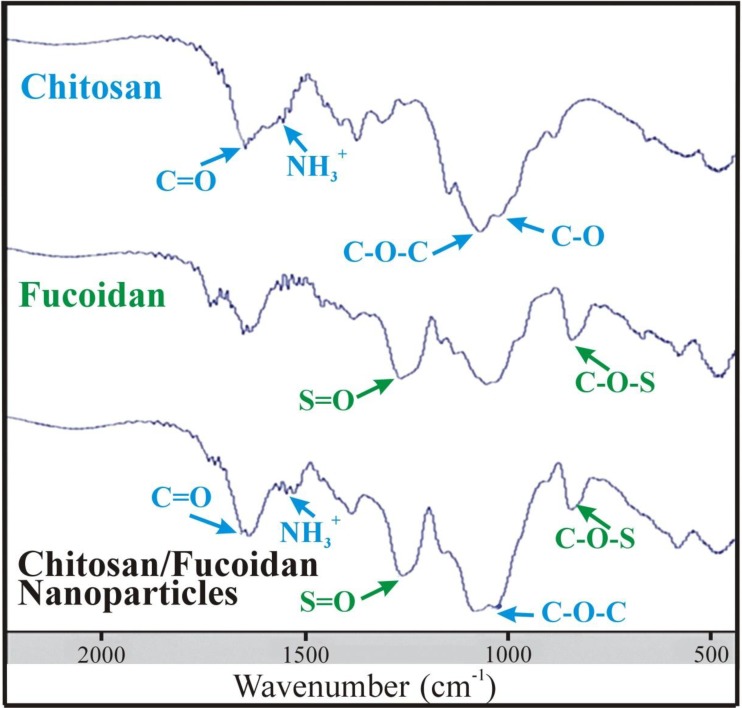
The FTIR spectra of CS, F, and CS/F NPs.

Figure 1 shows the FT-IR spectra of CS, fucoidan and CS/F NPs. The CS exhibited characteristic peaks of NH_3_^+^ (protonated amino group) bending vibrations and C=O (carbonyl group) stretching of the secondary amide at 1560 cm^−1^ and 1650 cm^−1^, respectively. The peaks at 1150 cm^−1^ and 1026 cm^−1^ indicated asymmetric C-O-C stretching and C-O skeletal vibration of CS [19]. The characteristic peaks of the fucoidan spectrum at 1160–1260 cm^−1^ and 845 cm^−1^ were associated with the S=O asymmetric stretching and C-O-S stretching of the sulfate groups [20]. In the CS/F NPs spectrum, both the characteristic peaks of CS and fucoidan were present, but a red shift of the C=O group of CS appeared, indicating possible changes in the environment of the group (Table 1) [35]. The distinctive peaks of the fucoidan spectrum were present at the same wavenumber, revealing that noncovalent interactions occurred between CS and fucoidan. The CS/F NPs were formed through an electrostatic interaction because of a positively charged amino group on the CS and a negatively charged sulfate group on the fucoidan under physiological conditions [19,36].

Table 2 presents the composition of each group of CS/F NPs. The characteristics of CS/F NPs in phosphate-buffered saline were shown in Table 3. The C3F1, C4F1, and C5F1 NPs exhibited comparable particle sizes as the pH values increased from 6.0 to 7.4. These results indicated that C3F1, C4F1, and C5F1 NPs remained stable and were insensitive to environmental pH changes. By contrast, the C1F1 and C2F1 NPs exhibited significant pH sensitive properties. The C1F1 NPs swelled considerably as the pH level rose, and discomposed at pH 7.4. The C2F1 NPs demonstrated the same trend as the C1F1 NPs, but swelled at a higher pH value (pH 7.2). The results can be explained based on the charge ratio of CS to fucoidan for each group (Table 2). The charge ratios of CS to fucoidan for the C5F1, C4F1, and C3F1 NPs are 5.26:1, 4.21:1, and 3.16:1, respectively. Although the pH value rose to 7.4, which was higher than the 6.5 pKa of CS, the excessive ammonium ions could still form stable electronic interactions with the sulfate groups [36]. In the C1F1 NPs, the CS and fucoidan exhibited comparable amounts of charges. As the pH value was higher than 6.5, the deionized ammonium ions caused the NPs to swell and disintegrate rapidly. In the C2F1 NPs, the positive CS charges were slightly higher than the negative fucoidan charges. As the pH value increased, the amount of positive charges was insufficient to form stable nanoparticles. Therefore, the C2F1 NPs were less sensitive to pH changes than the C1F1 NPs.

**Table 1:** Characteristic peaks of FTIR spectrum.

Compound	Characteristic Peaks
**Chitosan (CS)**	-NH_3_^+^ (1560 cm^−1^); -C=O (1650 cm^−1^); C-O-C (1150 cm^−1^); C-O (1026 cm^−1^)
**Fucoidan (F)**	S=O (1160–1260 cm^−1^); C-O-S (845 cm^−1^)
**CS/F NPs**	Both the characteristic peaks of CS and fucoidan were present, but a red shift of the C=O group of CS appeared.

**Table 2:** The composition of the prepared CS/F NPs. The CS and fucoidan used was 200 μL for each sample. The final pH value of the CS/F NPs solutions is 6.0.

Group	Chitosan (mg/mL, pH 6.0)	Fucoidan (mg/mL, pH 6.0)	Weight Ratio (CS:F)	Charge Ratio (CS:F)
C1F1	5	5	1:1	1.05:1
C2F1	10	5	2:1	2.11:1
C3F1	15	5	3:1	3.16:1
C4F1	20	5	4:1	4.21:1
C5F1	25	5	5:1	5.26:1

**Table 3:** The characteristics of CS/F NPs under phosphate-buffered saline, including particle size, zeta potential and PDI value.

	Particles Size (nm)							
	C1F1	C2F1		C3F1		C4F1	C5F1	
pH 6.0	372 ± 38	340 ± 5		331 ± 6		344 ± 2	326 ± 21	
pH 6.6	865 ± 129	331 ± 6		312 ± 2		327 ± 5	316 ± 3	
pH 7.0	1050 ± 149	334 ± 11		298 ± 8		330 ± 4	293 ± 10	
pH 7.2	1051 ± 124	429 ± 25		309 ± 4		324 ± 4	274 ± 6	
pH 7.4	1252 ± 114	747 ± 52		497 ± 54		348 ± 4	271 ± 6	
	Zeta potential (mV)							
	C1F1	C2F1		C3F1	C4F1		C5F1	
pH 6.0	8.0 ± 0.2	11.7 ± 0.8		13.8 ± 0.3	14.5 ± 0.6		13.2 ± 0.9	
pH 6.6	0.9 ± 2.8	7.8 ± 0.5		9.0 ± 0.7	10.4 ± 1.6		9.6 ± 0.4	
pH 7.0	−3.1 ± 2.7	4.1 ± 0.5		5.4 ± 0.8	6.1 ± 0.6		5.9 ± 0.5	
pH 7.2	−3.7 ± 1.5	3.3 ± 0.6		3.4 ± 0.1	3.9 ± 0.6		4.4 ± 0.7	
pH 7.4	−4.7 ± 1.3	0.7 ± 0.6		−1.1 ± 1.4	3.6 ± 3.1		1.8 ± 0.5	
	PDIs							
	C1F1	C2F1	C3F1			C4F1		C5F1
pH 6.0	0.24 ± 0.02	0.28 ± 0.01	0.31 ± 0.01			0.25 ± 0.02		0.35 ± 0.03
pH 6.6	0.29 ± 0.04	0.29 ± 0.01	0.27 ± 0.03			0.25 ± 0.01		0.37 ± 0.02
pH 7.0	0.37 ± 0.07	0.29 ± 0.03	0.27 ± 0.02			0.26 ± 0.01		0.32 ± 0.03
pH 7.2	0.33 ± 0.04	0.38 ± 0.03	0.30 ± 0.04			0.26 ± 0.01		0.29 ± 0.02
pH 7.4	0.41 ± 0.04	0.28 ± 0.03	0.28 ± 0.01			0.26 ± 0.02		0.24 ± 0.01

Data are mean ± SD of values calculated on 3 distinct batches (*n* = 3).

**Figure 2:**
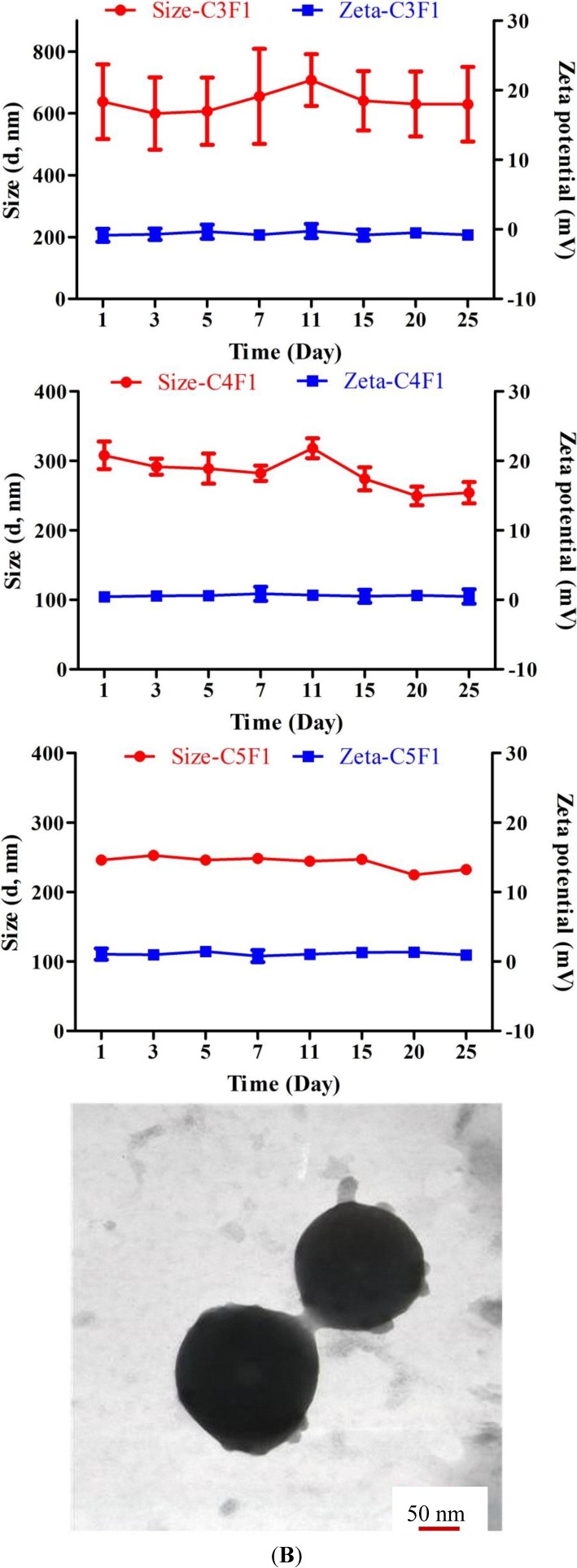
(**A**) Storage stability of C3F1, C4F1 and C5F1 NPs at 4 °C; (**B**) TEM image of C5F1 NPs in phosphate-buffered saline (pH 7.4).

The zeta potential tended to decrease as the pH changed from 6.0 to 7.4 in all groups (Table 3). The results were due to the number of positive ammonium ion decreasing as the pH increased. However, the C4F1 and C5F1 NPs still maintained a positive zeta potential at pH 7.4. The excessive amount of CS caused these results. Considering that NPs interact with cell populations along the respiratory tract containing negatively charged cell membranes, including mucus, epithelial cells, and macrophages, positively charged NPs are favorable [37]. The particle sizes of C3F1, C4F1, and C5F1 NPs were approximately 300 nm, 330 nm, and 270 nm, respectively, at pH 6.0 to pH 7.4 (Table 3). Nanosized particles have been reported to have an obstinate residency in the lungs [38] and an ability to evade phagocytosis and mucociliary clearance [39,40]. Moreover, nanoparticles exhibiting an approximate diameter of 250 nm are likely able to diffuse through lung mucus more easily than larger NP formulations can [41]. Brownian diffusion is the potential mechanism for the deposition of particles exhibiting a diameter of less than 500 nm and occurring in the acinar region of the lungs [42]. Figure 2A presents the storage stability of the C3F1, C4F1, and C5F1 NPs. The particle size variation of the C5F1 NPs was the least among all of the groups. The C5F1 NPs could maintain compactness and stability for 25 day. The C5F1 NPs in TEM images were spherical and exhibited diameters of approximately 230–250 nm (Figure 2B).

**Figure 3:**
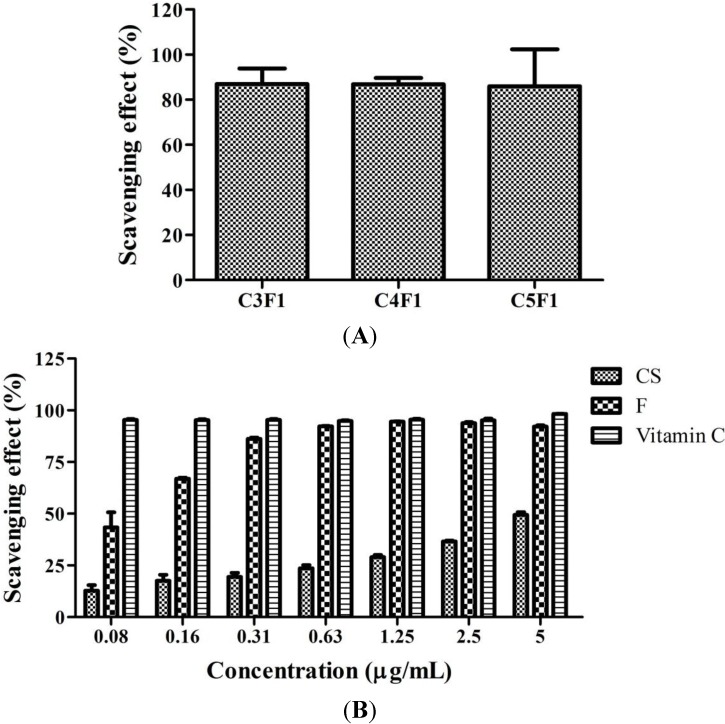
DPPH radical scavenging activity of (**A**) C3F1, C4F1, and C5F1 NPs and (**B**) CS, and F. Using vitamin C as positive control. Data are mean ± SD of values calculated on 3 distinct batches (*n* = 3).

### 2.2. DPPH Scavenging Activity of CS/F NPs

As shown in Figure 3A, the C3F1, C4F1, and C5F1 NPs demonstrated a DPPH scavenging activity of up to 80%. No significant difference among these NPs was observed. Furthermore, Figure 3B illustrates the concentration effect of CS and fucoidan on scavenging percentages to evaluate whether CS or fucoidan is responsible for DPPH scavenging activity. Using vitamin C as a positive control group, the DPPH scavenging effect was approximately 90% when the fucoidan concentration was higher than 0.31 mg/mL (Figure 3B). In addition, fucoidan presented a significantly higher scavenging effect than CS did. These results suggested that the DPPH scavenging effect of CS/F NPs primarily derives from fucoidan.

**Figure 4:**
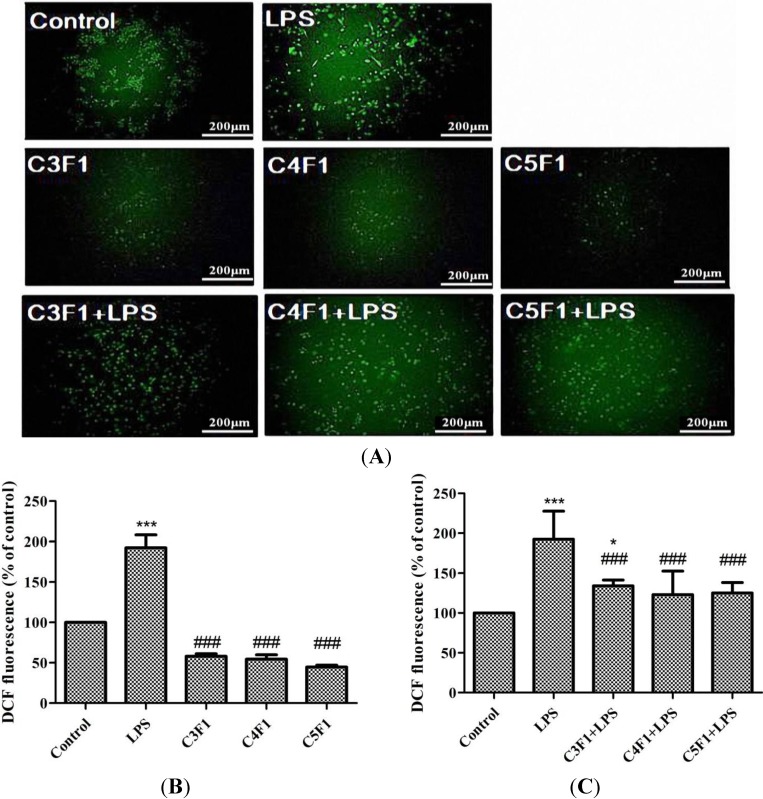
(**A**) ROS generation after exposure CS/F NPs to RAW 264.7 cells for 24 h. The intracellular ROS is observed by fluorescence microscope with green spots; (**B**) Effect of NPs on ROS production of RAW 264.7 and (**C**) LPS-induced RAW 264.7 cells detected by flow cytometry. Data are mean ± SD of values calculated on 5 distinct batches (*n* = 5). Statistical analysis was performed by one-way ANOVA. ** *p* < 0.01 *versus* control. *** *p* < 0.001 *versus* control. ^###^ *p* < 0.001 *versus* LPS.

### 2.3. Effects of CS/F NPs on ROS Production

Regarding the antioxidant activity test, the lipopolysaccharide (LPS)-induced intracellular ROS production against the RAW 264.7 cells was studied. The increased ROS after adding LPS was easily visualized using fluorescence microscopy, as shown in Figure 4A. Regarding quantification, the level of generated ROS was then determined using a flow cytometric analysis (Figure 4B,C). The ROS level increased in the LPS-induction group and was comparable with that of the control group in the C3F1, C4F1, and C5F1 groups (Figure 4B). Furthermore, C3F1, C4F1, and C5F1 NPs all significantly reduced the level of ROS induced by the LPS (Figure 4C). These results suggest that the CS/F NPs effectively reacted with ROS resulting in a reduced concentration.

**Figure 5:**
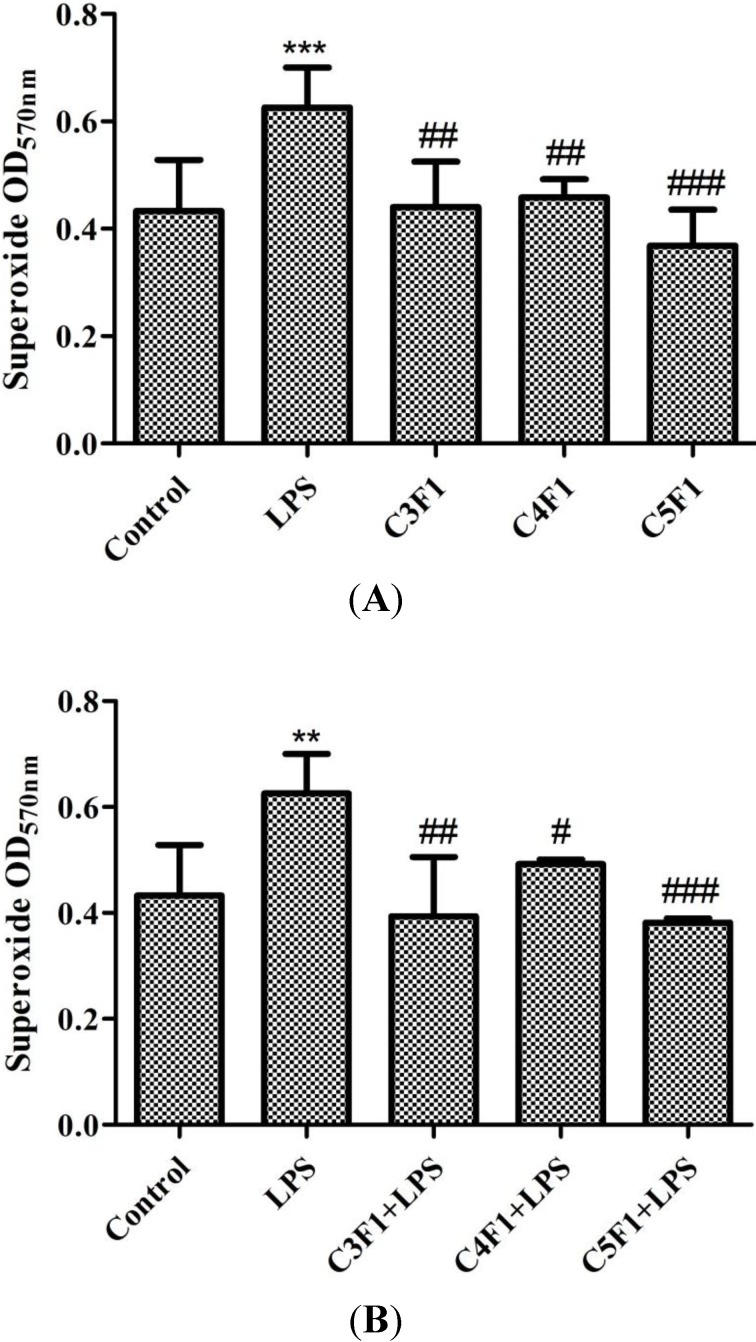
Effect of C3F1, C4F1, and C5F1 NPs on O_2_^−^ production of (**A**) RAW 264.7 cells and (**B**) LPS-induced RAW 264.7 cells. Data are mean ± SD of values calculated on 3 distinct batches (*n* = 3). Statistical analysis was performed by one-way ANOVA. ** *p* < 0.01 *versus* control, *** *p* < 0.001 *versus* control. ^#^ *p* < 0.05 *versus* LPS, ^##^ *p* < 0.01 *versus* LPS, ^###^ *p* < 0.001 *versus* LPS.

We also used the nitroblue tetrazolium (NBT) assay to monitor superoxide anion (O_2_^−^), which is an ROS frequently observed in various phagocytic cells. As shown in Figure 5A, the C3F1, C4F1, and C5F1 groups exerted no effect on O_2_^−^ production, but the LPS induced a significant O_2_^−^ level increase. Figure 5B shows that the O_2_^−^ level induced by the LPS decreased considerably after CS/F NPs were added. The effect was observed in the C3F1, C4F1, and C5F1 groups. According to the experimental results, the CS/F NPs exhibited an effective inhibitory effect on O_2_^−^ concentration.

Chitosan was reported to demonstrate antioxidant activities because of a strong hydrogen-donating ability [43]. Fucoidan obtained from *Fucus vesiculosus* exhibited excellent scavenging capacities on the hydroxyl and superoxide radicals [25,44]. The antioxidant ability of CS/F NPs revealed in our experiments was reasonable and corresponded with that of the aforementioned previous study. The CS/F NPs developed in the current study could be potential antioxidant carriers in a delivery system. Both chitosan and fucoidan play vital roles in the antioxidant property of CS/F NPs.

### 2.4. Anti-Inflammatory Effect of CS/F NPs

**Figure 6:**
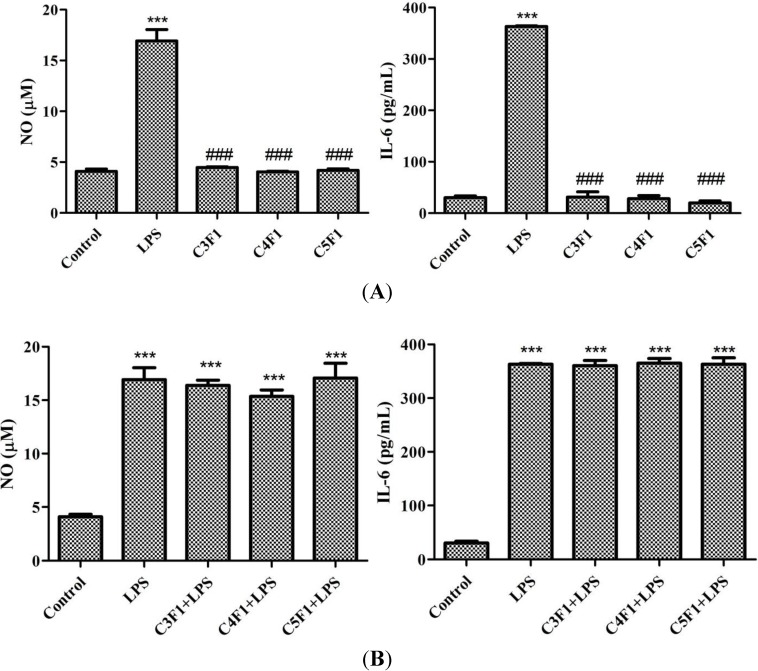
Effect of C3F1, C4F1, and C5F1 NPs on NO (**left** panel) and IL-6 (**right** panel) production of (**A**) RAW 264.7 cells and (**B**) LPS-induced RAW 264.7 cells. Data are mean ± SD of values calculated on 3 distinct batches (*n* = 3). Statistical analysis was performed by one-way ANOVA. *** *p* < 0.001 *versus* control. ^###^ *p* < 0.001 *versus* LPS.

LPS, a highly conserved cell wall component of gram-negative bacteria, is known to initiate signaling cascade for inflammatory mediator expression including cytokines, such as IL-6, and cytotoxic molecules, such as NO. Thus, NO and IL-6 are primary molecules involved in macrophage-mediated innate immune responses [45]. In the current study, LPS treatment caused a significant increase in NO and IL-6 production in RAW 264.7 cells (Figure 6). In the C3F1, C4F1, and C5F1 groups, the amount of NO and IL-6 was comparable with that of the control group (Figure 6A). As shown in Figure 6B, LPS-induced NO and IL-6 production caused no variation between the LPS and LPS + NP groups (including the LPS + C3F1, LPS + C4F1, and LPS + C5F1 groups). Based on the experimental results, the CS/F NPs activated no host immune cells into inflammation-mediated cytotoxic conditions induced by IL-6 production and NO generation.

### 2.5. Cytotoxicity of CS/F NPs

**Figure 7:**
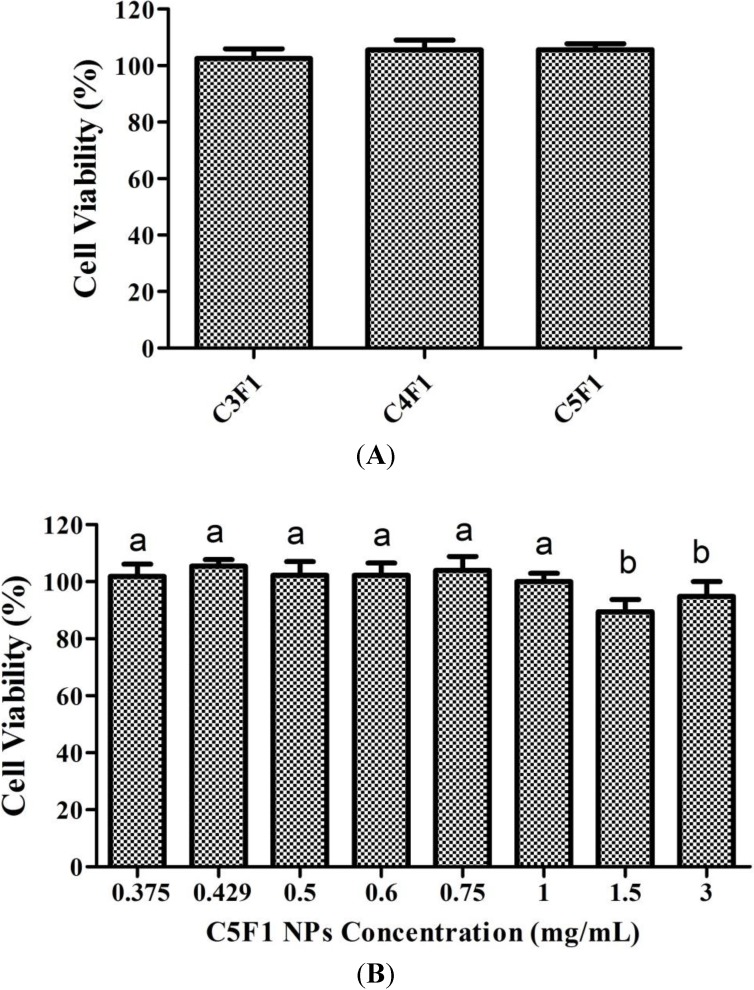
(**A**) Effect of C3F1, C4F1, and C5F1 NPs on A549 cell viability measured by MTT assays 24 h post-coculturing; (**B**) Effect of C5F1 NPs concentration on A549 cell viability measured by MTT assay 24 h post-coculturing. Data are mean ± SD of values calculated on 5 distinct batches (*n* = 5). Statistical analysis was performed by one-way ANOVA. Data with different letters express significant difference at *p* < 0.05.

This study also attempted to confirm the extent to which CS/F NPs affect cell viability by adding the CS/F NPs to an A549 cell culture. Figure 7A presents the cytotoxic effects of the CS/F NPs on the A549 cells by using MTT assay analyses. The cell viability was comparable in the C3F1, C4F1, and C5F1 NPs, which were 102.5%, 105.5%, and 105.6%, respectively. Furthermore, we also plotted the cell viability *versus* the concentration of the NPs (Figure 7B). The results indicated that cell viability was higher than 80% in the C5F1 NPs as its concentration was between 0.375 mg/mL and 3 mg/mL. The cytotoxicity effects of the C5F1 NPs on the A549 cells were exerted in a dose-independent manner.

### 2.6. In Vitro Release of Gentamicin (GM)

**Figure 8:**
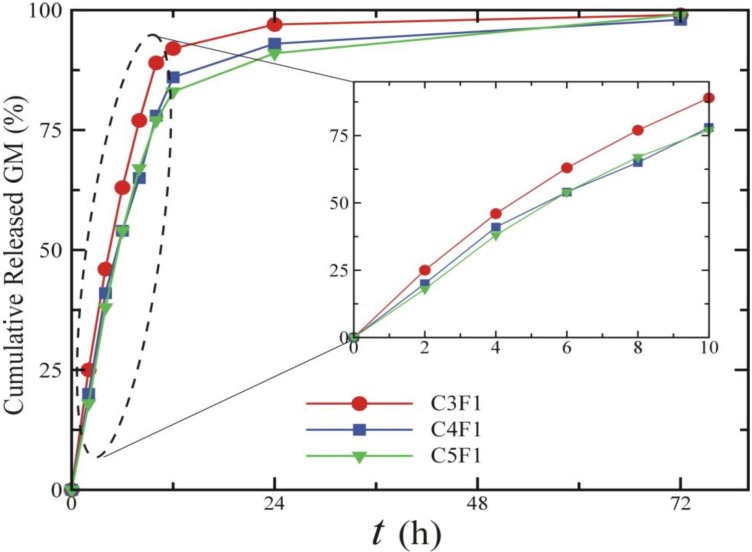
The release kinetics of GM from CS/F NPs.

In this study, GM is used as a model antibiotic for *in vitro* releasing test. The encapsulation efficiency of GM in CS/F NPs was 94%. Figure 8 plots the amount of GM released from the prepared NPs. The release of GM exhibited a biphasic profile. After an initial 10 h burst release of GM, the rate of release slowed, as reflected by the decreasing slope of the plot of cumulative amount of GM released against time. For the first 10 h, the linear curve exhibited a zero-order delivery of GM. After 72 h releasing, the cumulative percentage of GM released was about 99%, 98% and 99% in C3F1, C4F1 and C5F1 groups, respectively. The rapid releasing profile can be explained by reference to the hydrophilic property of GM. The dissolved GM could diffuse into the release medium easily. Moreover, GM on the surface of NPs might contribute to speedy releasing. The slow releasing curve in the second phase is due to polymer degradation [46]. GM is an aminoglycoside antibiotic widely used for treating many types of bacterial infections in airway inflammatory diseases, particularly those caused by Gram-negative bacteria. The biphasic releasing of GM is effective for bacterial growth inhibition. In the initial stage, rapid release of GM could inhibit bacterial growth efficiently; in the second stage, sustained releasing of GM could suppress bacterial infection continuously. Briefly, CS/F NPs prepared in this study are potential carriers for antibiotics delivery.

Fucoidan mimics certain biological activities of heparin, including anticoagulant and growth factor binding properties [47,48]. Heparin and fucoidan have been reported to bind and dimerize stromal cell-derived factor-1(SDF-1) [49] and to enhance the basic fibroblast growth factor (bFGF) induced tube formation of endothelial cells [50]. Numerous studies have focused on heparin in pharmacology and biomaterial science; however, the feasibility of using fucoidan in biomaterials and delivery systems remains limited. Fucoidan derived from brown algae is highly promising for use in clinical settings as an alternative to heparin obtained from animals, and thus further studies on this aspect of fucoidan use are warranted.

## 3. Experimental Section

Chitosan (deacetylation degree ≥ 75%) and fucoidan (from *F. vesiculosus*) were supplied by Sigma Chemical Co. (St. Louis, MO, USA). Human A549 lung cells (BCRC 60074) and Murine RAW 264.7 macrophages (BCRC 60001) were both obtained from the Bioresource Collection and Research Center, Hsinchu, Taiwan. All of the other chemicals were of reagent grade and were purchased from Sigma Chemical Co. unless stated otherwise.

### 3.1. Preparation of CS/F NPs

A simple polyelectrolyte self-assembly method was used in this study to prepare the CS/F NPs by performing ultrasonication at room temperature. To increase solubility, the CS was treated with H_2_O_2_ to produce low-molecular-weight CS (38 kDa) by using our previously developed method [20]. The NPs with various CS-to-fucoidan weight ratios, including 1:1, 2:1, 3:1, 4:1, and 5:1 (named the C1F1, C2F1, C3F1, C4F1 and C5F1 groups, respectively), were prepared by adding a 200 μL CS/acetic acid solution (pH 6.0) at varying concentrations (5 mg/mL, 10 mg/mL, 15 mg/mL, 20 mg/mL, and 25 mg/mL) using a pipette into a 200 μL aqueous fucoidan solution (5 mg/mL, pH 6.0) under probe-type ultrasonicating (pulse-on 3 s and pulse-off 7 s; total 30 s) at ice bath. The self-assembled nanoparticles were collected using centrifugation at 14,000 rpm for 5 min. The final pH value of the CS/F NPs solutions is 6.0. The supernatants were removed, and the NPs were lyophilized or resuspended in phosphate buffered saline for further study.

### 3.2. Characterization of CS/F NPs

FT-IR (Brucker Tensor 27) was used to analyze the peak variation of amino and sulfate groups on the prepared NPs. A Zetasizer nano ZS (Malvern Instruments Ltd., Worcestershire, UK) was used to measure the size distribution, zeta potential, and polydispersity index (PDI) of CS/F NPs. We used TEM (JEOL, Tokyo, Japan) for observing morphology. To prepare the TEM sample, a drop of the NP suspension was applied to a 200-mesh copper grid for approximately 10 min. A filter paper was then taped onto the grid to remove surface water, and the NP was fixed in 1% formaldehyde for 2–3 h. The sample was then positively stained using an alkaline bismuth solution [19,51].

### 3.3. Cell Culture

For the A549 cells, the culture medium used was an F-12K medium (Gibco, Grand Island, NY, USA) supplemented with 10% FBS, 1.5 g/L sodium bicarbonate, 100 units/mL penicillin, and 100 μg/mL streptomycin. For the RAW 264.7 cells, the culture medium was Dulbecco’s modified Eagle’s medium (DMEM) (Gibco, Grand Island, NY, USA) supplemented with 10% FBS, 2.5 g sodium bicarbonate, 3.7 g HEPES, 100 units/mL penicillin, and 100 μg/mL streptomycin. The cultures were maintained at 37 °C in a humidified atmosphere containing 5% carbon dioxide.

### 3.4. DPPH Scavenging Test

Chitosan and fucoidan were diluted in ddH_2_O for each experimental concentration (0–5 mg/mL). Subsequently, 50 μL of 0.2 mM 1,1-diphenyl-2-picrylhydrazyl (DPPH) solution was added to a 50 μL CS or fucoidan solution and incubated at room temperature for 45 min in the dark. The absorbance was measured using a SpectraMax 340PC^384^ microplate spectrophotometer (Molecular Devices, Sunnyvale, CA, USA) at 517 nm. Vitamin C was used as a positive control [52]. Scavenging activity was calculated using the following formula:

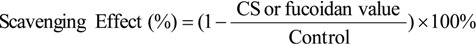
(1)


The results of each concentration were calculated on 3 distinct batches.

### 3.5. Reactive Oxygen Species Determination

The intracellular ROS was determined using 2′,7′-dichlorofluorescin diacetate (DCFH-DA) [52] and NBT reduction assay [53]. The DCFH-DA enters the cell where it reacts with ROS to form the highly fluorescent compound dichlorofluorescein (DCF). Briefly, RAW 264.7 cells were planted in 12-well plates at a density of 2.0 × 10^5^ cells/well and allowed to attach for 24 h before treatment. After the cells were exposed to CS/F NPs and LPS for 24 h, the medium was changed to a serum-free DMEM containing 20 μM of DCFH-DA and incubated for 30 min at 37 °C. The cells were then washed once with PBS, collected using 0.25% trypsinization, rewashed with PBS, and then resuspended in 1 mL of PBS. The cells were observed using an inverted fluorescence microscope (Olympus IX71, Tokyo, Japan). The fluorescence was then determined at 488 nm excitation and 525 nm emission by using a Cytomics FC500 flow cytometry system (BD Biosciences Aria, Franklin Lakes, NJ, USA).

Regarding the NBT reduction analysis, RAW 264.7 cells (1.0 × 10^6^ cells/well) were planted in 6-well plates and allowed to attach for 24 h before treatment. After the cells were exposed to CS/F NPs and LPS for 24 h, the cells were harvested using centrifugation and suspended in 300 μL of NBT solution for 1 h. Subsequently, 50 μL of 2 N HCl was added to terminate the reaction. After centrifugation, 200 μL of dimethylsulfoxide was added to the cell pellets to solubilize the formazan deposits. The amount of formazan formed was assayed spectrophotometrically at 570 nm by using a spectrophotometer (SpectraMax 340PC^384^, Molecular Devices, Sunnyvale, CA, USA).

### 3.6. Nitric Oxide Assay

RAW 264.7 cells at a density of 3 × 10^4^ cells/well were planted in a 24-well plate and preincubated with CS/F NPs and/or LPS for 24 h. Subsequently, 50 μL of 1% sulfanilamide was added to 100 μL of supernatant for 10 min before adding 50 μL of 0.1% *N*-(1-naphthyl)-ethylenediamine dihydrochloride for another 10 min. The absorbance of the product dye was measured at 540 nm by using a spectrophotometer (SpectraMax 340PC^384^, Molecular Devices, Sunnyvale, CA, USA).

### 3.7. Interleukin-6 Assay

RAW 264.7 cells (3 × 10^4^ cells/well) were planted in a 24-well plate and preincubated with CS/F NPs and LPS for 24 h. Subsequently, 100 μL of supernatant were extracted to quantify interleukin-6 (IL-6), a proinflammatory mediator, using an enzyme-linked immunosorbent assay (ELISA). LPS was used as a positive control to stimulate the IL-6 and validate the ELISA protocol. The principle of the ELISA is based on the sandwich technique. ELISA plates (NUNC, Polylabo, Strasbourg, France) were coated with capture antibodies (200 ng/mL) and blocked using bovine serum albumin (1% *w/v*) for 2 h at room temperature. After the 100 μL diluted samples were added to the ELISA plates, a secondary antibody was added to the 96-well ELISA plate at a concentration of 250 ng/mL, and the plates were incubated at room temperature for 2 h. Streptavidin-conjugated horseradish peroxidase was then added to the plates. The substrate solution was subsequently added and the solution obtained thereby was incubated for 20 min. The enzyme reaction was terminated with the addition of a 2 N H_2_SO_4_ solution. The absorbance of the samples was read at 450 nm using an ELISA plate reader (SpectraMax 340PC^384^, Molecular Devices, Sunnyvale, CA, USA).

### 3.8. MTT Assay

Cell viability was measured using an MTT (3-(4,5-dimethylthiazol-2-yl)-2,5-diphenyltetrazolium bromide) assay (Sigma-Aldrich, St. Louis, MO, USA) according to manufacturer instructions. The A549 cells (1.0 × 10^4^ cells/well) were planted in 96-well plates and allowed to attach for 24 h before exposure to CS/F NPs solutions for another 24 h. The MTT (0.5 mg/mL) was then added to each well and incubated at 37 °C for 4 h. After cautiously aspirating the culture medium, 1 mL of dimethyl sulfoxide (DMSO) was added and thoroughly mixed for 10 min. Absorbance was determined at 570 and 630 nm (SpectraMax 340PC^384^, Molecular Devices, Sunnyvale, CA, USA). Cells grown without NPs were used as the control. Cell viability was expressed as a percentage of that observed in the control specimens.

### 3.9. Preparation of GM-Loaded CS/F NPs and in Vitro Release

For preparing GM-loaded CS/F NPs, 200 μL CS/acetic acid solution (15 mg/mL, 20 mg/mL and 25 mg/mL) was mixed homogeneously with a 200 μL 1 mg/mL GM solution. Aqueous fucoidan solution (5 mg/mL) were then added to the CS/GM mixture by flush mixing, using a pipette tip under ultrasonic vibration at ice bath. Next, the prepared NPs were collected by centrifugation at 14,000 rpm for 5 min. The NPs were then lyophilized or resuspended in phosphate buffered saline for further study.

In the release studies, GM was released from CS/F NPs. The prepared GM-loaded CS/F NPs were suspended in 1 mL of phosphate-buffered saline (PBS) and then incubated at 37 °C with agitation (100 r.p.m., Distek-2230A, North Brunswick, NJ, USA). At specified intervals, 500 μL supernatant of sample was extracted and the solution was replenished with fresh buffer. The amount of GM in the supernatant was quantified by an indirect spectrophotometric method, using *o*-phtaldialdehyde as derivatizing agent [54]. The procedure is as follows. After dissolving 0.25 g *o*-phtaldialdehyde in a mixture of methanol (6.25 mL) and 2-mercaptoethanol (0.3 mL), the resulting solution was mixed with 0.04 M sodium borate (56 mL, pH 8) solution. For the colorimetric measurement, GM solution, *o*-phtaldialdehyde reagent and isopropanol were mixed in an equal volume ratio, and incubated for 30 min at room temperature. The absorbance of the samples was read at 340 nm using a spectrophotometer (SpectraMax 340PC^384^, Molecular Devices, Sunnyvale, CA, USA). The amount of GM was determined from a calibration curve based on known concentrations of GM.

### 3.10. Statistical Analysis

All quantitative data were expressed as mean ± standard deviation (SD). A one-way ANOVA and post test statistical analyses were performed using GraphPad Prism 5. A *p* value of <0.05 indicated statistical significance.

## 4. Conclusions

To achieve a sustained local effect of a drug in the lungs, developing a carrier with excellent biocompatibility, biodegradability, antioxidant activity and no inflammatory response is crucial when frequent administration is necessary. In addition, the carriers must be able to prevent mucociliary clearance and phagocytic mechanisms. To address this challenge, we developed a novel CS/F NP and evaluated its potency for a pulmonary delivery system. The prepared CS/F NPs were formed by electrostatic interaction and proved stable in phosphate-buffered saline (pH 6.0–7.4). The physicochemical properties of CS/F NPs, such as size, zeta potential, and stability, depend on the weight ratio of chitosan to fucoidan. In the *in vitro* tests conducted in this study, the CS/F NPs exerted no cytotoxic effects on the A549 cells, as the concentration of NPs was between 0.375 mg/mL and 3 mg/mL. The CS/F NPs exhibited scavenging effects and antioxidant activities according to the DPPH assay and ROS determination tests. Furthermore, undesirable inflammatory reactions were not induced. The CS/F NPs controlled the release of GM with an initial burst effect followed by a slow drug release. Overall, the experimental results indicate promising features of CS/F NPs for use in a pulmonary drug delivery system.
